# Supplementary material for: Immune disease risk variants regulate gene expression dynamics during CD4+ T cell activation
Source: Nat Genet. Author manuscript; Available in PMC 2022 Jun 18. (PMC9197762; doi:10.1038/s41588-022-01066-3)
Supplement: Supplementary Material [file EMS144055-supplement-Supplementary_Material.pdf]

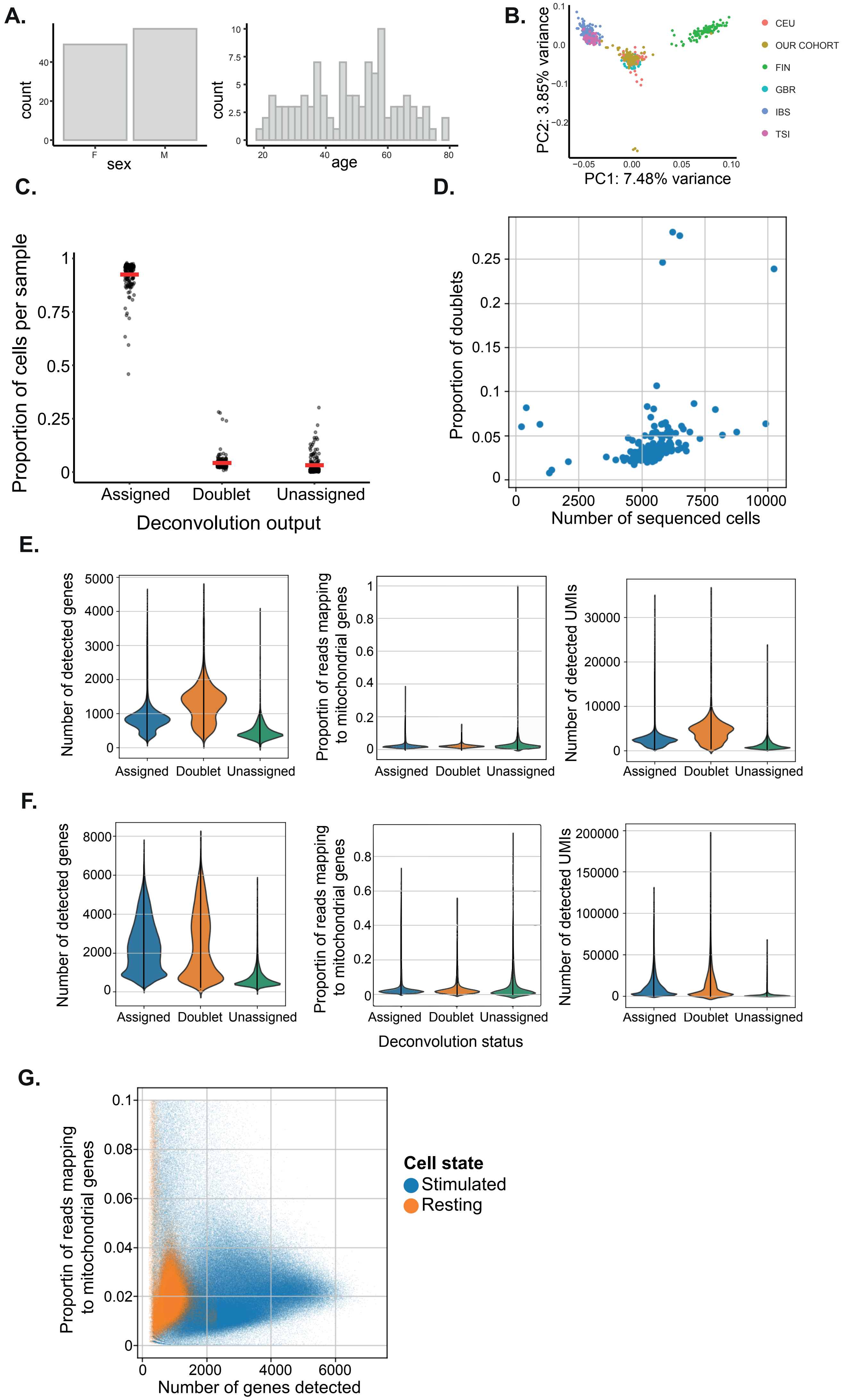

Supplementary Figure 1

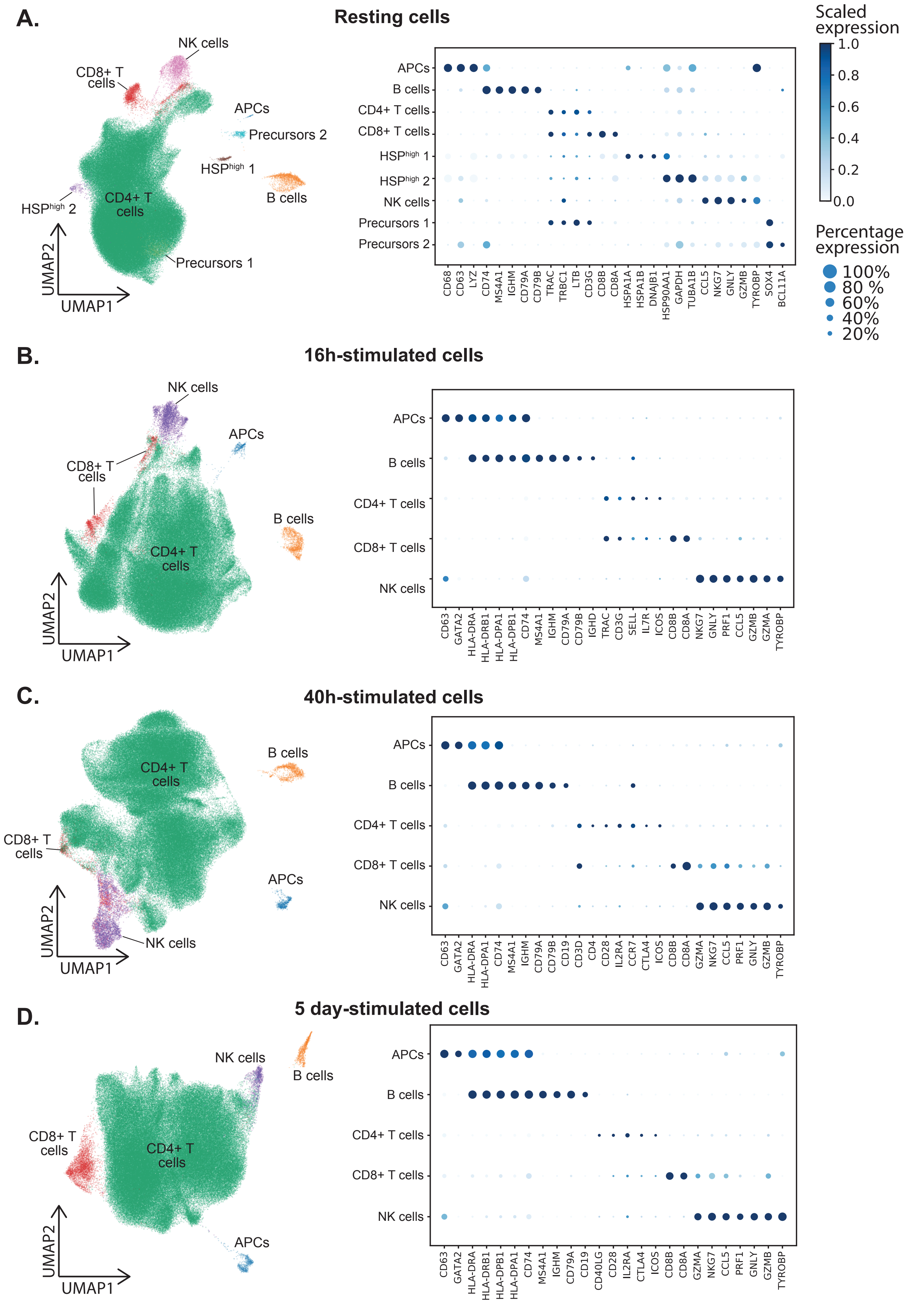

Supplementary Figure 2

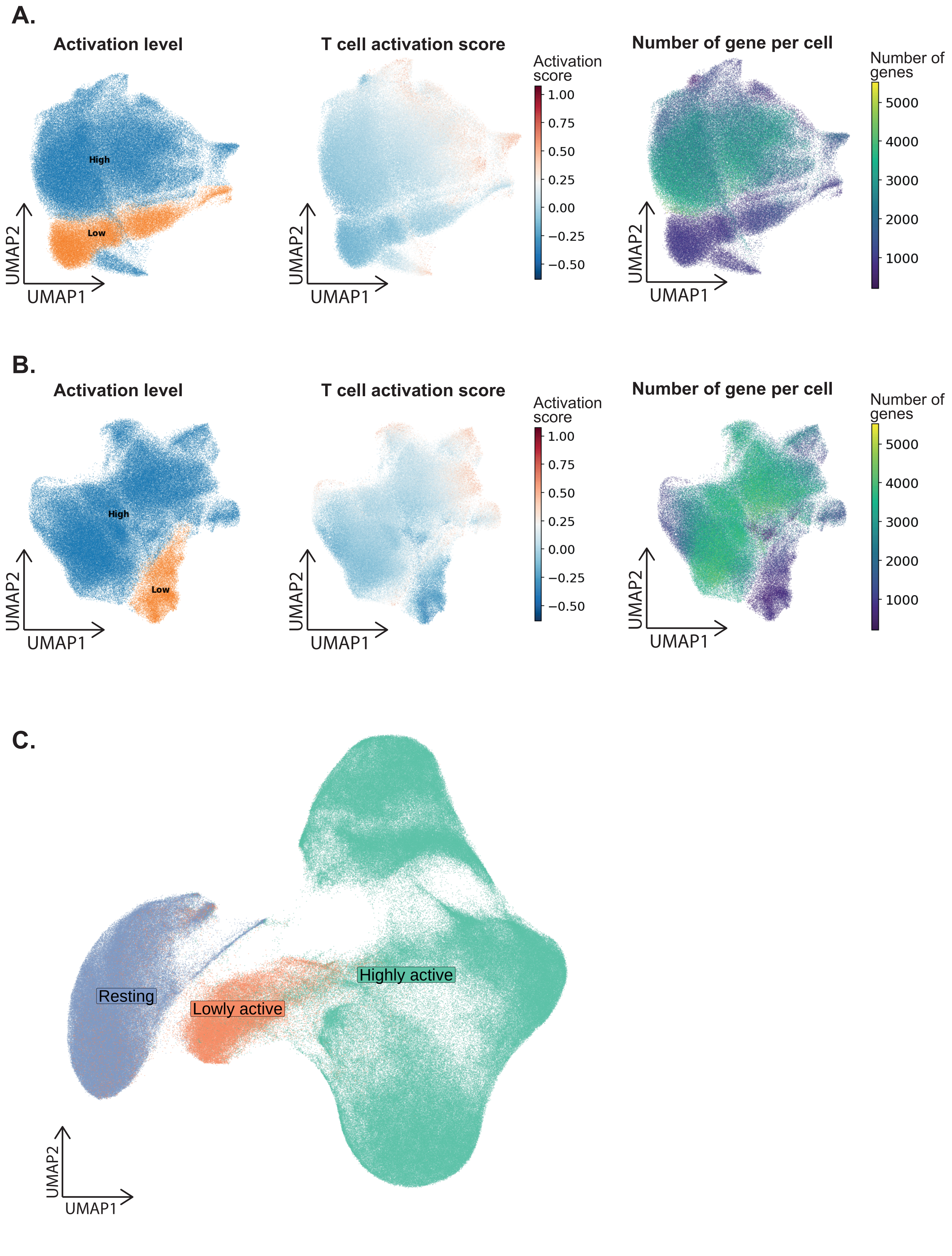

Supplementary Figure 3

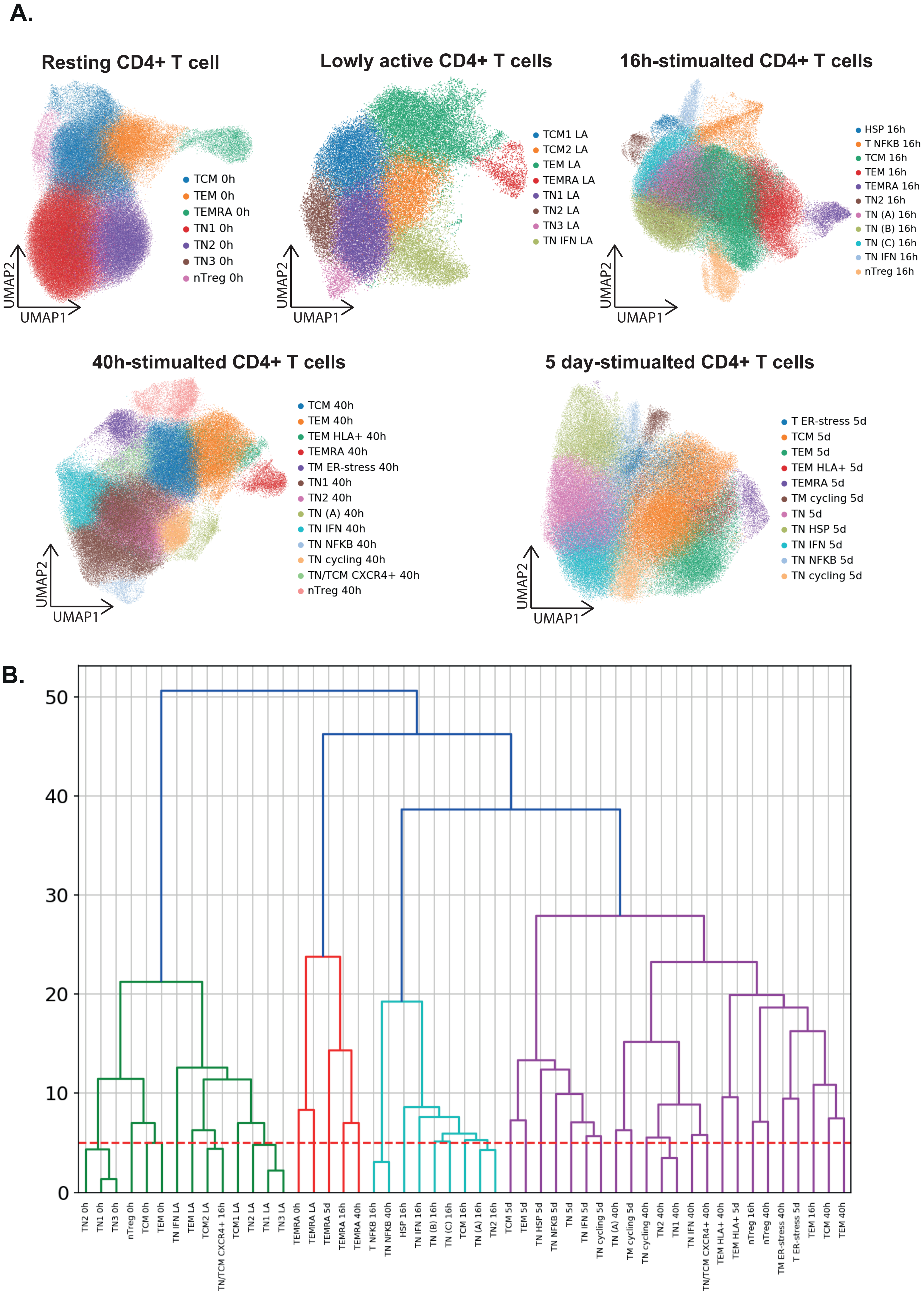

Supplementary Figure 4

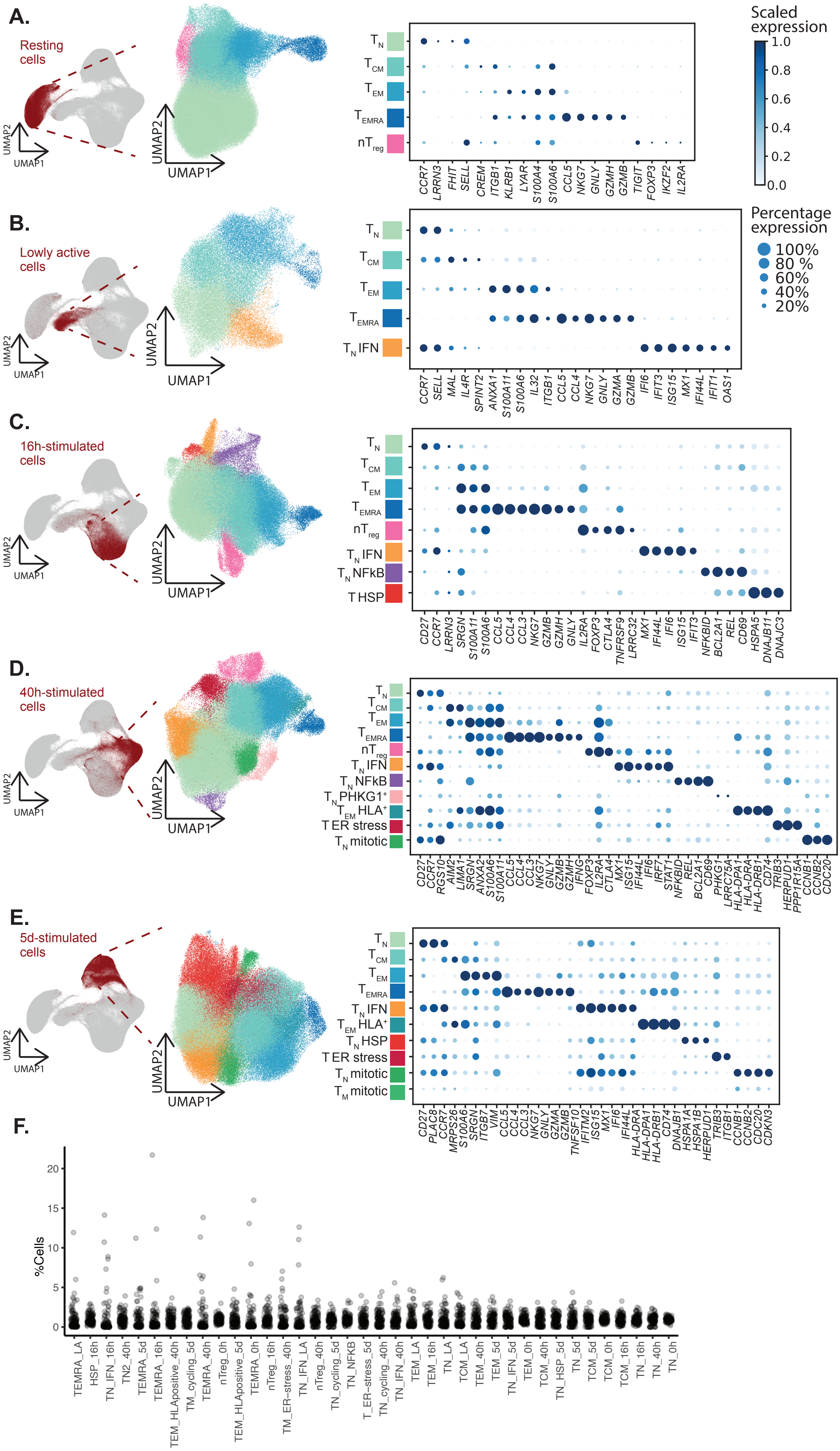

Supplementary Figure 5

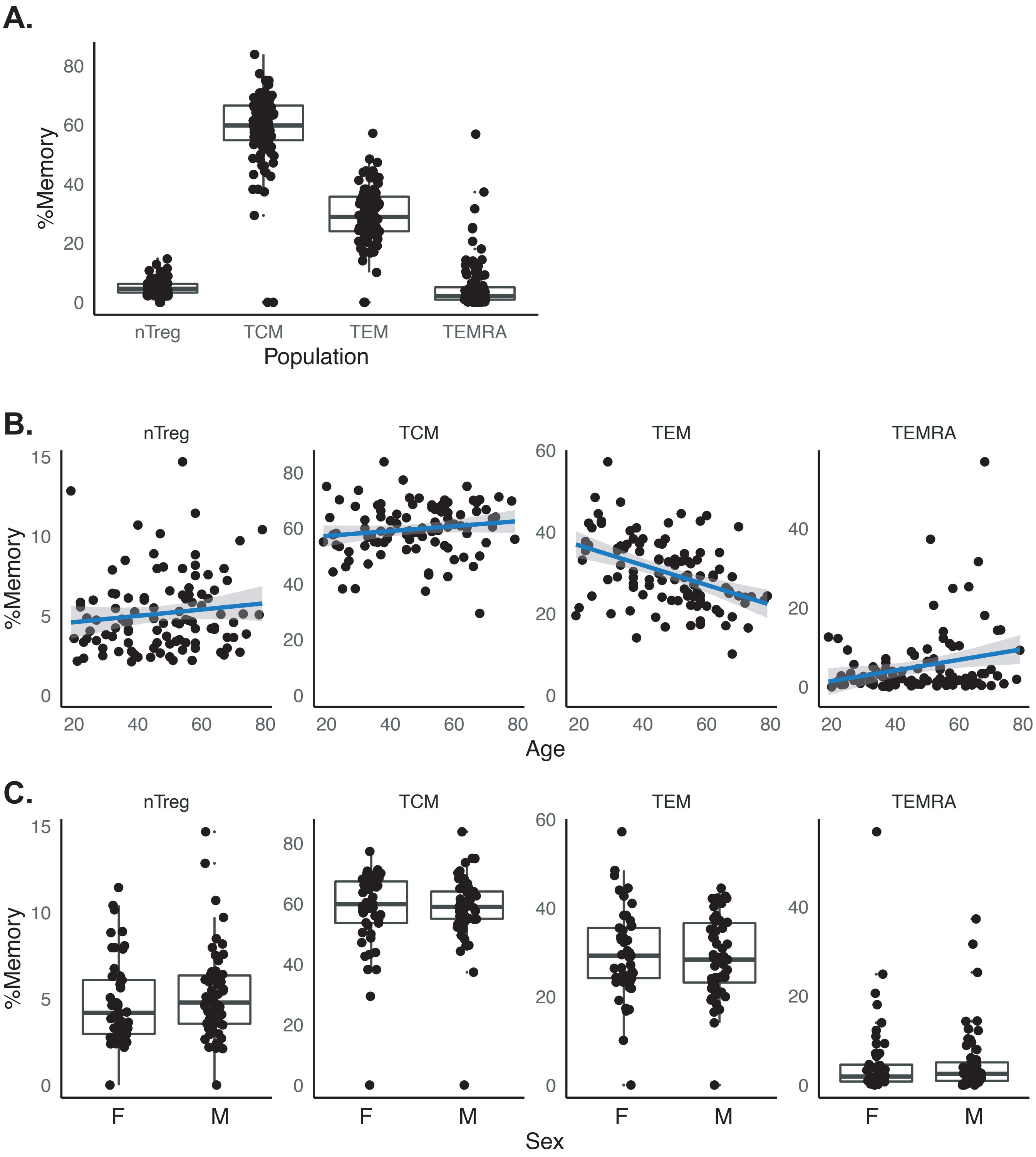

Supplementary Figure 6

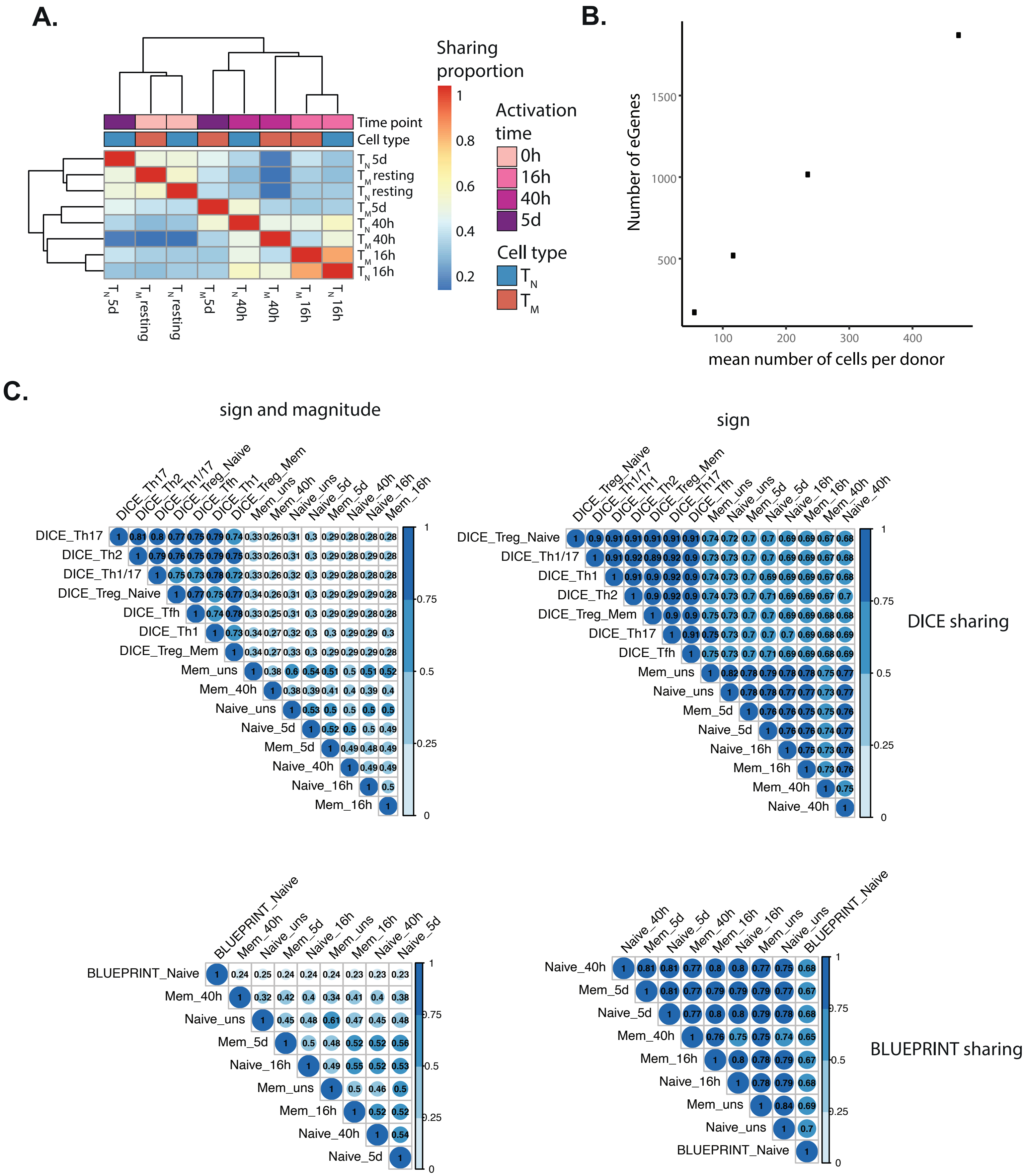

Supplementary Figure 7

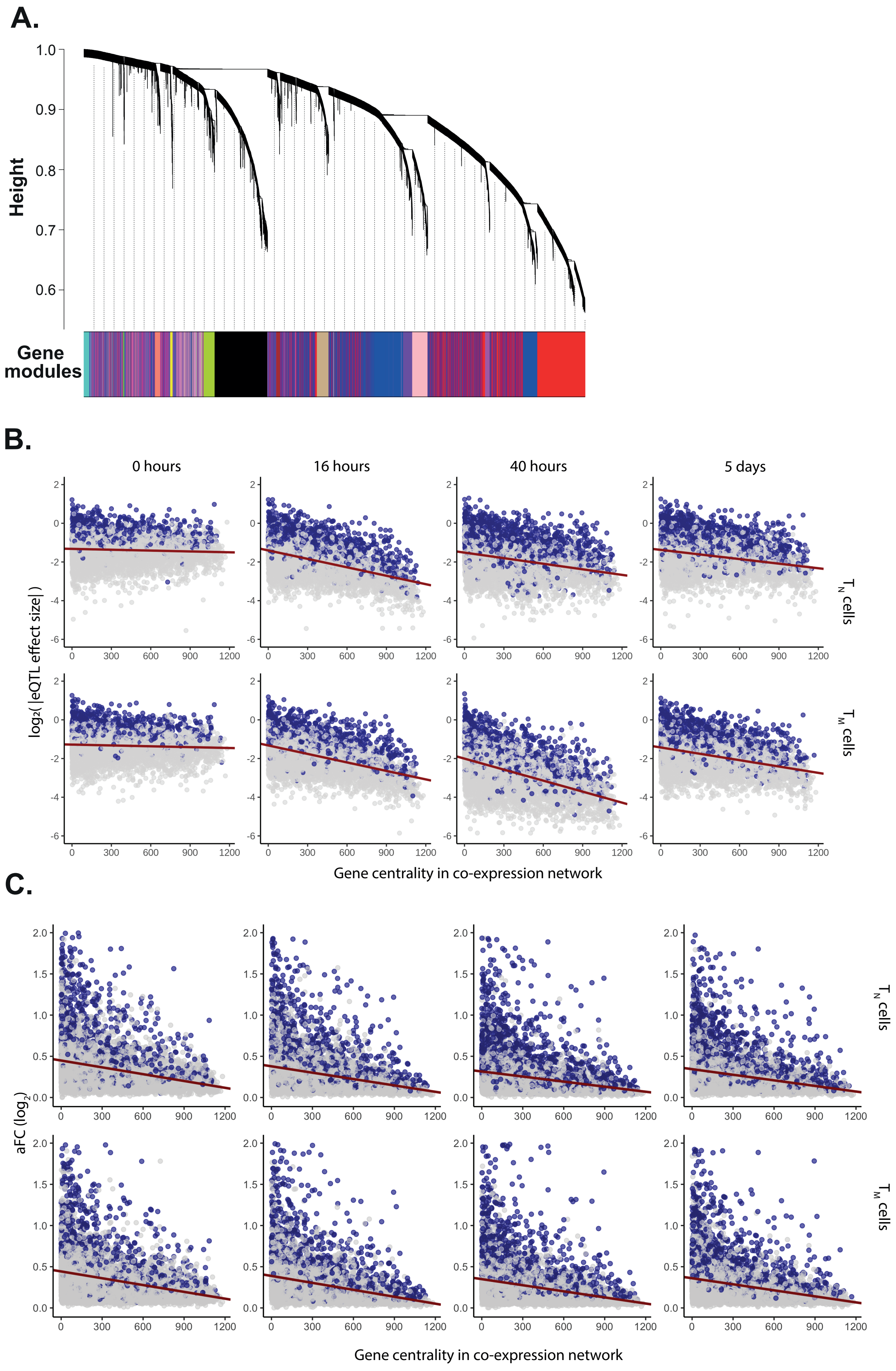

Supplementary Figure 8

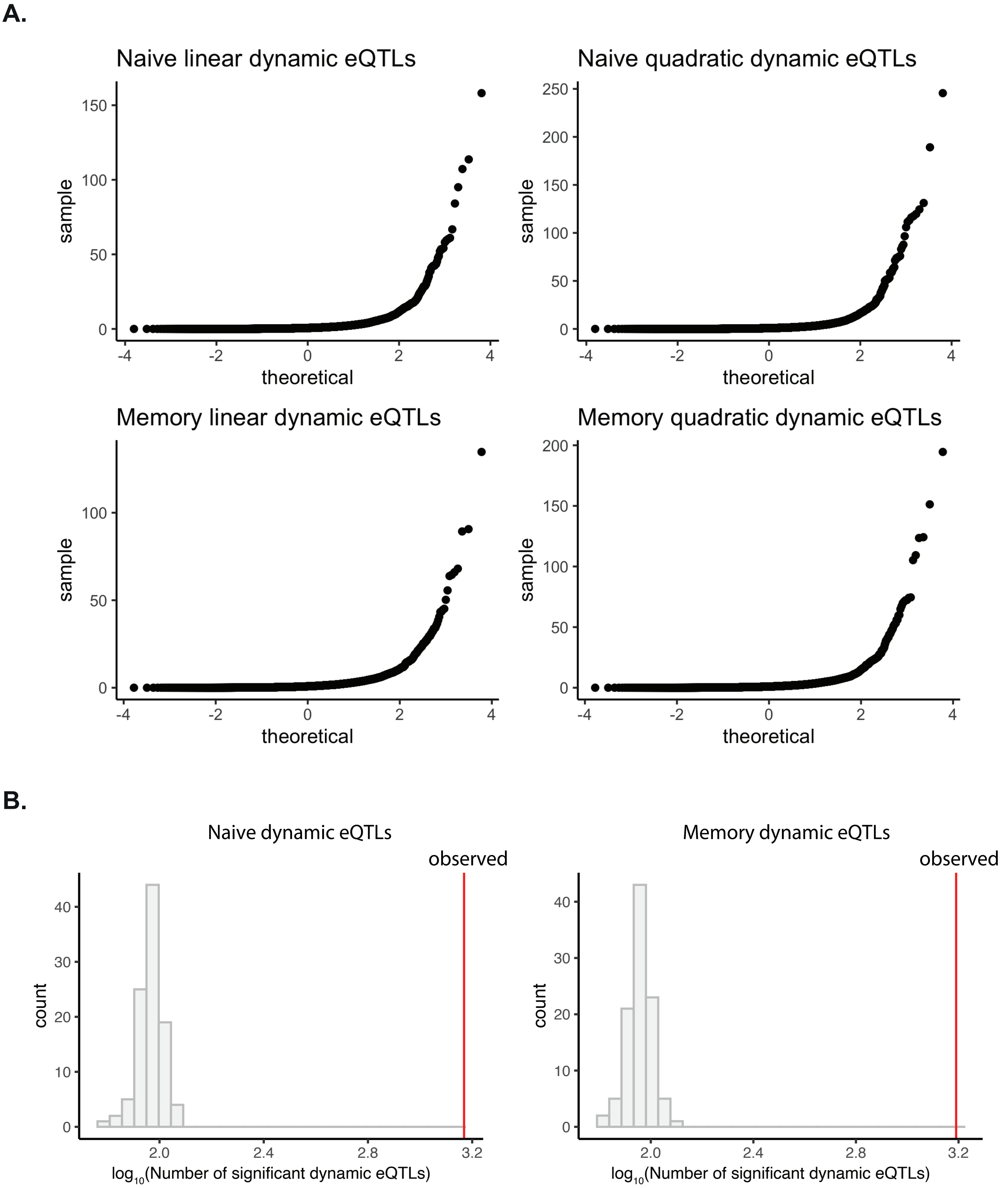

Supplementary Figure 9

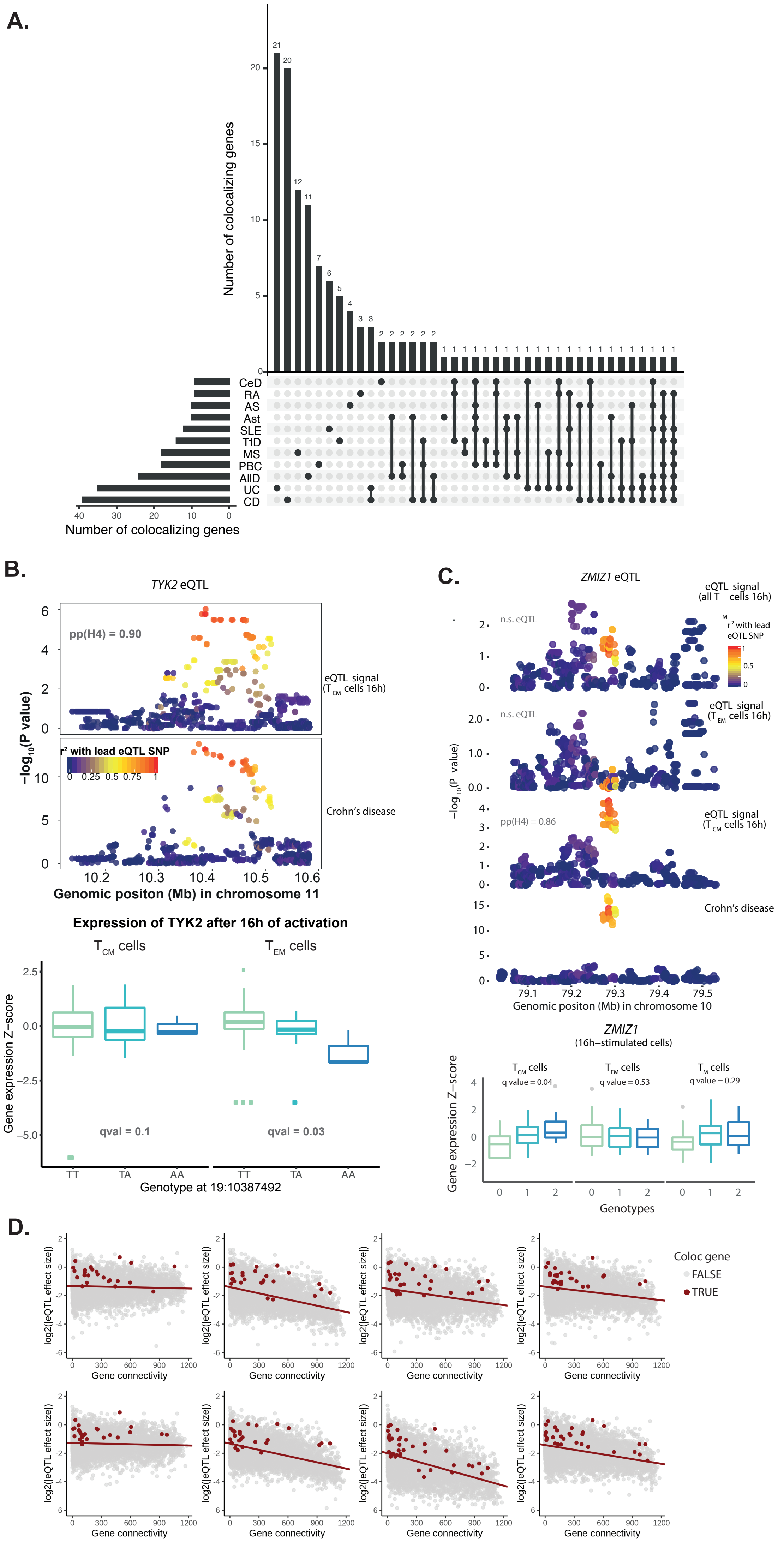

Supplementary Figure 10

Supplementary figure legend

Supplementary Figure 1. Cohort and quality metrics of the scRNA-seq data set. A) Distriution of sex and age for all individuals. B) Genetic ancestry was inferred for each individual in the cohort by comparing their genotypes (common genetic variants obtained from genotyping and imputation) with a panel of five well-defined European ancestries in PCA space. The populations in this panel were derived from the 1000G project (Methods) and corresponded to Utah residents with Northern and Western European ancestry (CEU), Finnish in Finland (FIN), British in England and Scortland (GBR), Iberian Population in Spain (IBS), and Toscani in Italy (TSI). C) Proportion of cells per sample which are confidently assigned to one individual (assigned), contain RNA from more than one individual (doublets) or are unassigned (unassigned). D**)** Dot plot showing the relationship between number of cells per inlet and number of doublets. E**-F)** Violin plots showing the number of detected genes (left), proportion of reads mapping to mitochondrial genes (central) and number of detected UMIs (right) in assigned cells, doublets and unsigned cells. These metrics are shown separately for resting (E) and stimulated (F) samples. G**)** Dot plot showing the number of detected genes and proportion of reads mapping to mitochondrial genes in each cell. Colours indicate resting (orange) and stimulated (blue) cells.

Supplementary Figure 2. Identification and removal of cellular contaminations in the scRNA-seq data set. Separate UMAP embeddings for cells obtained at four time points. Colours represent cell populations derived from unsupervised clustering. Green cells are CD4+ T cells. Other colours represent contaminations that were removed for subsequent analyses. **A)** Resting cells. **B)** 16h-stimulated cells. **C)** 40h-stimulated cells. **D)** 5 day-stimulated cells.

Supplementary Figure 3. Identification of a population of lowly active CD4+ T cells. A-B) UMAP embedding of all CD4+ T cells sampled at 16h (**A**) and 40h (**B**) after activation. Each dot is a cell, with colours indicating either the lowly active population (orange; left panel), the scaled average expression of a set of T cell activation markers from bulk RNA-seq (central panel), or the number of genes detected per cell (right panel). **C)** Location of the lowly active population of cells in a UMAP embedding containing CD4+ T cells from all time points in the study.

Supplementary Figure 4. Cluster merging strategy. A) Unsupervised clustering was applied independently to the five cell groups of cells identified in the study (resting, lowly

active, 16 hours, 40 hours, and five days). Colours represent cell populations derived from unsupervised clustering. This resulted in 51 cell clusters. **B)** The similarity of clusters was assessed by performing PCA and estimating the Euclidean distance between pairs of clusters based on the first 100 principal components. Below the dotted red line are clusters with high levels of similarity that were merged together This resulted in 38 distinct groups of cells.

Supplementary Figure 5. CD4+ T cell subpopulation markers. A) Cell subpopulations and marker genes detected independently for CD4+ T cells belonging to each of five broad cell groups: resting (**A**), lowly active (**B**), 16h-stimulated (**C**), 40h-stimulated (**D**), and 5 day-stimulated (**E**) cells. UMAP embeddings (left panels) show each cell as one dot, with colours indicating cell subpopulations derived from unsupervised clustering and cluster merging (38 subpopulations in total). Equivalent subpopulations detected at more than one time point are indicated in the same colour. Dot plots (right panels) show the top marker genes identified for each subpopulation. Shades of blue indicate the scaled mean expression of a gene in each subpopulation, while dot sizes correspond to the proportion of cells in the subpopulation that express the gene. **F)** Dot plot showing the percentage of cells belonging to an individual within each cluster. The X-axis is arranged by cluster size (the smallest to the largest).

Supplementary Figure 6. Memory T cell distribution. A) Percentage of memory T cell subpopulations. Each dot represents an estimate obtained from a separate individual. Boxes extend from the 25th to the 75th percentiles, with central lines indicating the median. Boxplot whiskers further extend by ±1.5 times the interquartile range from the limits of each box. N of biologically independent samples: 106. **B)** Relationship between age and percentage of nTregs, T_{CM}, T_{EM} and T_{EMRA}. Each dot represents a measurement obtained from a separate individual. Lines indicate the best linear fit and shaded areas show 95% confidence intervals obtained from the respective linear models. **C)** Percentage of memory T cell subpopulations stratified by sex. Each dot represents a measurement obtained from a separate individual. Boxes extend from the 25th to the 75th percentiles, with central lines indicating the median. Boxplot whiskers further extend by ±1.5 times the interquartile range from the limits of each box. N of biologically independent samples: 106.

Supplementary Figure 7. eQTL sharing. A) eQTL sharing was assessed by comparing the effect sizes and directions of eQTLs across cell types (naive and memory cells) and time points using mashR (**Methods**). This heatmap indicates the proportion of eQTLs shared by sign and magnitude between cell type and time point combinations. **B)** The relationship between mean number of cells per donor and power to detect eGenes. T_{CM} cluster was

subsampled three times. **C)** Overlaps of eQTLs with DICE and BLUEPRINT were assessed by comparing the effect sizes and directions of eQTLs across cell types (naive and memory cells) and time points using mashR.

Supplementary Figure 8. Identification of gene expression patterns active throughout CD4+ T cell activation. A) Genes were ordered into a co-expression matrix using WGCNA (Methods). This dendrogram shows genes arranged by similarity. Modules of genes with correlated expression are indicated by blocks of colour at the bottom. **B)** Relationship between a gene's connectivity (as inferred from co-expression network analysis) and the effect size of its lead eQTL signal. All eQTL effect sizes were log-transformed. Blue dots represent significant eGenes, while gray dots represent genes which do not pass the eQTL multiple test correction. **C)** Relationship between a gene's connectivity and the allelic fold change (aFC) of its lead eQTL signal. All eQTL effect sizes were log-transformed. Blue dots represent significant eGenes, while gray dots represent genes which do not pass the eQTL multiple test correction.

Supplementary Figure 9. Dynamic eQTL discovery A) Q-Q plots of all SNP-gene pairs that are tested in the dynamic model. **B)** Distribution of the number of significant dynamic eQTLs in the permuted data.

Supplementary Figure 10. eGenes colocalizing at immune disease loci. A) Number of colocalizing genes shared between different immune traits. Bar plots indicate the number of genes in each set. **B and C)** Locus plots for a colocalization between a *TYK2* **(B)** and *ZMIZ1* **(C)** and a GWAS signal for Crohn's disease. Each dot corresponds to a genetic variant, with colours indicating their LD with the lead eQTL variant. Box plots indicate the expression level of *TYK2* **(B)** and *ZMIZ1* **(C)**, stratified by genotype. Each dot corresponds to a measurement from a different individual. Boxes extend from the 25th to the 75th percentiles, with central lines indicating the median. Boxplot whiskers further extend by ±1.5 times the interquartile range from the limits of each box. (B) N of biologically independent samples: TCM TYK2: 100, TEM TYK2: 97 (C) N of biologically independent samples: TCM ZMIZ1: 100, TEM ZMIZ1: 97, TM ZMIZ1: 95. P-values were derived using tensorQTL and corrected as described in Methods.P-values were derived using tensorQTL and corrected as described in Methods. **D)** Relationship between a gene's connectivity (as inferred from co-expression network analysis) and the effect size of its lead eQTL signal. Red dots represent colocalizing genes and gray dots represent all other genes.

Supplementary notes

Genotyping data analysis and imputation

Quality control was performed by removing samples with <95% called genotypes, as well as keeping only variants with MAF > 5%, SNP call rate > 95%, and in Hardy-Weinberg equilibrium (HWE; p-value < 0.001).

Imputation of untyped variants was performed as described in Bossini-Castillo et al[51]. Briefly we used BEAGLE 4.1 [52] with a reference panel consisting of the 1000 Genomes Phase 3[53] and the UK10K samples[54]. Variants derived from imputation were quality filtered using the following parameters: allelic R-squared (AR2) >= 0.8, HWE p-value < 0.001, and MAF > 10%. In total we retained 4,641,747 variants after imputation, which were used for eQTL analysis. We confirmed that all but one of the individuals in our cohort clustered with the British in England and Scotland (GBR) population in PCA space (**Supplementary Figure 1B**). This individual clustered with the Finnish population and was removed from any further analyses. Finally, related individuals were kept for every analysis except eQTL mapping, where one random individual from each pair was used.

Co-expression network analysis

In order to preserve most of the resolution provided by scRNA-seq while also minimising zero-inflation and technical noise, gene expression was aggregated per individual and per cluster into a pseudobulk count matrix. More specifically, raw UMI counts per gene were summed across all cells belonging to the same cluster and to the same individual. Summed expression values were normalised by library size and gene length, resulting in a single expression matrix with transcript per million (TPM) units (i.e. a pseudobulk matrix). The resulting pseudobulk matrix was imported into R (v3.6.1) and analysed using the weighted gene co-expression network analysis (WGCNA) package (v1.69)[28]. Only genes with ≥ 1 TPM in at least 30 samples were used for this analysis. TPM values were first log-transformed, after which unwanted sources of variation (i.e. cell culture batch, reported sex, and age) were regressed out using limma's

(v3.40.6) removeBatchEffect() function[60]. Next, genes were filtered by their level of variability, with only genes showing a standard deviation ≥ 0.1 across samples being kept. This resulted in 11,130 genes taken forward for network construction.

The functions available in WGCNA were used to calculate network properties for these genes, such as their level of connectivity, as well as to build an adjacency matrix. The soft power parameter was set to 4 in these analyses, based on an evaluation of the [0,20] parameter space. The resulting adjacency matrix was transformed into Topological Overlap Matrices (TOM) of similarity and dissimilarity. Finally, hierarchical clustering was applied to the TOM dissimilarity matrix in order to build a dendrogram of genes. Gene modules were inferred from this dendrogram using R's dynamicTreeCut package (v1.63.1). Mean module expression values were calculated as the average expression of all genes belonging to that module in a given sample.

Pathway enrichment analysis

All pathway enrichment analyses were performed using gprofiler2 (v 0.1.9)[64], setting the gene list of interest as an unordered query and using all genes detected in the study as the background. Only enriched terms derived from the Kyoto Encyclopedia of Genes and Genomes (KEGG)[65] or the REACTOME database[66] were kept. gprofiler2's built-in approach (gSCS) was used for multiple testing correction. Enriched pathways were visualized in R using the pheatmap package (v1.0.12).

Analysis of eQTL sharing across cell types

To assess the sharing between eQTLs, we performed a meta-analysis across cell types and cell states using the multivariate adaptive shrinkage (mashR) method[25]. MashR is a Bayesian method which estimates the pairwise level of sharing between cell states, where an eQTL is defined as shared if it has the same effect size (within a factor of 0.5) and direction in two cell states. Furthermore, to identify shared eQTL effects between our dataset and DICE and BLUEPRINT datasets we analysed gene-variant pairs available in summary statistics of all $CD4_+$ T cells datasets using MashR. We identified the lead variant for each gene and we randomly selected four variants

per gene as the random set to fit the mashr mode.

Estimation of pairwise LD

We performed LD calculations based on the individual-level genotype information for the individuals in this study obtained from genotyping. Namely, we used PLINK (v1.90b4)[71] to calculate the correlation between pairs of genetic variants across all individuals in the cohort, using either the --r or the --r2 flags. We restricted this calculation to variants with MAF > 0.01, computing correlations for any pairs of variants located within 1 Mb of each other and at most 500 variants away. The command used was: *plink --r/--r2 --ld-window 500 --ld-window-kb 1000 --maf 0.01 --vcf-half-call h .*

Pre-processing of GWAS summary statistics

Full summary statistics files from previous GWAS studies were downloaded from the GWAS catalogue[72]. These files corresponded to a recent release containing harmonised summary statistics, which were lifted over to build GRCh38 of the genome and for which the effect size and effect direction of each variant was aligned to the reported alternative allele[33]. Any signals coming from the X or Y chromosomes, as well as from the MHC region (ch6:28,510,120 – chr6:33,480,577) were discarded. These summary statistics corresponded to 13 immune-mediated diseases: celiac disease[73], rheumatoid arthritis[74], ankylosing spondylitis[75], asthma[76], systemic lupus erythematosus[77], type 1 diabetes[78], multiple sclerosis[79], primary biliary cirrhosis[80], allergic disease[81], juvenile idiopathic arthritis[82], psoriasis[83], ulcerative colitis, and Crohn’s disease[84].
