# Supplementary material for: Immune disease risk variants regulate gene expression dynamics during CD4+ T cell activation
Source: Nat Genet. 2022 May 26;54(6):817–26. doi: 10.1038/s41588-022-01066-3 (PMC9197762; doi:10.1038/s41588-022-01066-3)
Supplement: Supplementary file 2 [file 41588_2022_1066_MOESM2_ESM.pdf]

nature portfolio

Corresponding author(s): Gosia Trynka

Last updated by author(s): Mar 1, 2022

Reporting Summary

Nature Portfolio wishes to improve the reproducibility of the work that we publish. This form provides structure for consistency and transparency in reporting. For further information on Nature Portfolio policies, see our Editorial Policies and the Editorial Policy Checklist.

Statistics

For all statistical analyses, confirm that the following items are present in the figure legend, table legend, main text, or Methods section.

n/a	Confirmed	
☐	☒	The exact sample size (*n*) for each experimental group/condition, given as a discrete number and unit of measurement
☐	☒	A statement on whether measurements were taken from distinct samples or whether the same sample was measured repeatedly
☐	☒	The statistical test(s) used AND whether they are one- or two-sided<br>*Only common tests should be described solely by name; describe more complex techniques in the Methods section.*
☐	☒	A description of all covariates tested
☐	☒	A description of any assumptions or corrections, such as tests of normality and adjustment for multiple comparisons
☐	☒	A full description of the statistical parameters including central tendency (e.g. means) or other basic estimates (e.g. regression coefficient) AND variation (e.g. standard deviation) or associated estimates of uncertainty (e.g. confidence intervals)
☐	☒	For null hypothesis testing, the test statistic (e.g. *F*, *t*, *r*) with confidence intervals, effect sizes, degrees of freedom and *P* value noted<br>*Give P values as exact values whenever suitable.*
☐	☒	For Bayesian analysis, information on the choice of priors and Markov chain Monte Carlo settings
☒	☐	For hierarchical and complex designs, identification of the appropriate level for tests and full reporting of outcomes
☐	☒	Estimates of effect sizes (e.g. Cohen's *d*, Pearson's *r*), indicating how they were calculated

Our web collection on statistics for biologists contains articles on many of the points above.

Software and code

Policy information about availability of computer code

Data collection: No software was used for data collecti

Data analysis

Imputation was performed using BEAGLE 4.1 with a reference panel consisting of the 1000 Genomes Phase 3 and the UK10K samples. LD calculations were performed on the individual-level genotype using PLINK (v1.90b4).
Raw scRNA-seq data were processed using the Cell Ranger Single-Cell Software Suite (v3.0.0, 10X-Genomics). Reads were first assigned to cells and then aligned to the human genome using STAR v2.5.1b with the hg38 build. Ensembl (v93) was used as a reference for gene annotation. Results from RNA quantification were imported into Python (v3.8.1) and analysed using scanpy (v1.4.4).
To assign cells to their respective individuals, a list of common exonic variants was compiled from the 1000 genomes project phase 3 exome-sequencing data. CellSNP (v0.99) was used to generate pileups at the genomic location of these variants. These pileups, in combination with the variants called from genotyping in each individual, was used as an input for Vireo (v1).
Unsupervised clustering was applied based on kNN graphs using the Leiden algorithm. The similarity of clusters to each other was assessed by performing PCA and estimating the Euclidean distance between pairs of clusters based on the first 100 principal components. Clusters with high levels of similarity or overlapping biological characteristics were merged together.
To perform trajectory inference, raw gene expression measurements were imported into R (v3.6.1) and analysed using monocle3 (v0.2.0).
For co-expression analysis, we used WGCNA package (v1.69). Gene modules were inferred from this dendrogram using R's dynamicTreeCut package (v1.63.1).
All pathway enrichment analyses were performed using gprofiler2 (v 0.1.9), setting the gene list of interest as an unordered query and using all genes detected in the study as the background. Enriched pathways were visualized in R using the pheatmap package (v1.0.12).
To identify cis-eQTLs we used tensorQTL (v1.0.3). To assess the sharing between eQTLs, we performed a meta-analysis across cell types and cell states using the multivariate adaptive shrinkage (mashR) method. For dynamic eQTL mapping, models were implemented in R using the lmer() function.
Coloc (v4.0.4) was used to test for colocalization between the eQTL and the GWAS signals.

For manuscripts utilizing custom algorithms or software that are central to the research but not yet described in published literature, software must be made available to editors and reviewers. We strongly encourage code deposition in a community repository (e.g. GitHub). See the Nature Portfolio guidelines for submitting code & software for further information.

Data

Policy information about availability of data

All manuscripts must include a data availability statement. This statement should provide the following information, where applicable:

- Accession codes, unique identifiers, or web links for publicly available datasets
- A description of any restrictions on data availability
- For clinical datasets or third party data, please ensure that the statement adheres to our policy

The raw single-cell RNA-sequencing data study is deposited in the European Genome-Phenome Archive (EGA), accession number EGAD00001008197. Genotypes are deposited in the EGA, accession number EGAD00010002291. Processed single cell data and summary statistics are available at https://trynkalab.sanger.ac.uk. Summary statistics from previously performed GWAS studies for 13 immune-mediated diseases were downloaded from the GWAS catalogue.
To compare the findings from our study with publicly available CD4+ T cell eQTLs we used eQTLs from Chen L. et al. 2016 (PMID: 27863251 ftp://ftp.ebi.ac.uk/pub/databases/blueprint/blueprint_Epivar//qtl_as/QTL_RESULTS/) and Schmiedel, B. J. et al. 2018 (PMID: 30449622 https://dice-database.org/downloads#eqtl_download).

Field-specific reporting

Please select the one below that is the best fit for your research. If you are not sure, read the appropriate sections before making your selection.

☒ Life sciences ☐ Behavioural & social sciences ☐ Ecological, evolutionary & environmental sciences

For a reference copy of the document with all sections, see nature.com/documents/nr-reporting-summary-flat.pdf

Life sciences study design

All studies must disclose on these points even when the disclosure is negative.

Sample size

Sample size was based on results from previous human eQTL studies (e.g. PMID: 29022597, 33664506), where 100-200 samples provide sufficient power to map eQTL.

Data exclusions

Variants derived from imputation were quality filtered using the following parameters: allelic R-squared (AR2) >= 0.8, HWE p-value < 0.001, and MAF > 10%.
Samples with less than 70% of reads mapping to cells were discarded. In addition, any cells with less than 200 detected genes, an unusually high number of genes (defined as over four standard deviations above the mean number of detected genes), or more than 10% of reads mapping to mitochondrial genes were removed from the data set. Any genes detected in less than 10 cells were discarded. Any cells labelled as "unassigned" (less than 0.9 posterior probability of belonging to any individual) or "doublets" (containing mixed genotypes) by Vireo were discarded. Finally, cell contaminations were removed from the data set, resulting in 655,349 (91.86% of total) high quality cells kept and successfully annotated as CD4+ T cells.
In co-expression analysis, only genes with ≥ 1 TPM in at least 30 samples were used. In addition, genes were filtered by their level of variability, with only genes showing a standard deviation ≥ 0.1 across samples being kept. This resulted in 11,130 genes taken forward for network construction.
To ensure the high quality eQTL mapping, we kept genes with non-zero expression in at least 10% of donors and mean count per million (cmp) higher than one. We retained between 8,940 and 11,516 genes. We also removed related individuals.
In GWAS studies, any signals coming from the X or Y chromosomes, as well as from the MHC region (ch6:28,510,120 – chr6:33,480,577) were discarded.
For each trait-cell type pair, we applied colocalization to any locus where a lead variant for a significant eQTL (q value < 0.1) was located

within 100 kb and in high LD (r2 > 0.5) with a significant GWAS variant. In addition, we required at least 50 variants to be available for testing at each candidate locus.

Replication	We used previously reported results from bulk RNAseq data and replicated markers of clusters. We also replicated that T cells form a continuum (Cano-Gamez et al. 2020; Kiner et al. 2021) and demonstrated that this continuum can be taken into account when mapping response eQTLs (as previously shown in different cell types by Strober et al. 2019; Cuomo et al. 2020). There is no single cell RNA-seq data available that would be suitable for replication of activation eQTLs.<br>No other experiments were performed in this study.
Randomization	Donors were allocated to experimental batches at random.
Blinding	No blinding was applied, as all samples were processed by the same operators.

Reporting for specific materials, systems and methods

We require information from authors about some types of materials, experimental systems and methods used in many studies. Here, indicate whether each material, system or method listed is relevant to your study. If you are not sure if a list item applies to your research, read the appropriate section before selecting a response.

Materials & experimental systems

n/a	Involved in the study	
☒	☐	Antibodies
☒	☐	Eukaryotic cell lines
☒	☐	Palaeontology and archaeology
☒	☐	Animals and other organisms
☐	☒	Human research participants
☒	☐	Clinical data
☒	☐	Dual use research of concern

Methods

n/a	Involved in the study	
☒	☐	ChIP-seq
☒	☐	Flow cytometry
☒	☐	MRI-based neuroimaging

Human research participants

Policy information about studies involving human research participants

Population characteristics	Blood samples were obtained from 119 healthy individuals of British ancestry. Of these, 67 were male (53.7%) and 52 female (56.3%), and the mean age of the cohort was 47 years (sd = 15.61 years).
Recruitment	Participants were recruited from GSK blood resource at Addenbrooke's Hospital. Blood was processed within 3h of collection. To our knowledge there was no selection bias.
Ethics oversight	Human biological samples were sourced ethically and their research use was in accord with the terms of informed consent under an IRB/EC approved protocol (15/NW/0282). Ethics was approved by Wellcome Sanger Institute ethicks committee.

Note that full information on the approval of the study protocol must also be provided in the manuscript.
